# A Global Analysis of the Complex Structural Organization of KCTD Proteins and Their Functional Implications

**DOI:** 10.3390/ijms27135745

**Published:** 2026-06-25

**Authors:** Nicole Balasco, Luciana Esposito, Simone Di Micco, Luigi Vitagliano

**Affiliations:** 1 Institute of Molecular Biology and Pathology (IBPM), CNR c/o Department of Chemistry, Sapienza University of Rome, Piazzale A. Moro 5, 00185 Rome, Italy; 2 Institute of Biostructures and Bioimaging (IBB), CNR, Via P. Castellino 111, 80131 Naples, Italy; luciana.esposito@cnr.it; 3 European Biomedical Research Institute of Salerno (EBRIS), Via S. De Renzi 50, 84125 Salerno, Italy; s.dimicco@ebris.eu

**Keywords:** AlphaFold, structural biology, protein–protein interactions, disease-associated mutations, hetero-oligomers

## Abstract

KCTD proteins exhibit significant structural complexity, arising from their modular organization, oligomerization, and intricate biological partnerships. Although their biological importance has been assessed for two decades, the biochemical basis of their activities is only partially understood. This is certainly due to the limited structural information that was available until a few years ago. Fortunately, some recent insightful structural studies and the advent of machine-learning-based approaches are rapidly changing the scenario. By surveying the literature and structural databases and integrating this information with ad hoc 3D-structure predictions, we provide a detailed view of the structural biology of these proteins at different levels: individual domains, full-length oligomers, functional hetero-oligomers formed by different family members, and complexes with functional partners. Collectively, these surveys and analyses provide insights into the family’s evolutionary history and its structure–function relationships. The family-wide coverage of structural information also indicates the extent to which structural similarities are reflected in functional analogies. Finally, the potential functional implications of the intricate architecture of these multimeric proteins and the tendency of their members to hetero-oligomerize are discussed from a functional perspective.

## 1. KCTD Proteins and Their Molecular Organizations

Proteins are fundamental players in all biological processes. Although these biomolecules may operate in highly dynamic states, structural complexity is often their hallmark of functional activity. This complexity is achieved by juxtaposing folded modules (domains) within the polypeptide chain, self-association (oligomerization), and functional interactions with partners. The occurrence or non-occurrence of these complexity-increasing events within a single protein family is an effective means of achieving functional specificity among its members. A prototypical example in this context is the KCTD (K)-potassium Channel Tetramerization Domain-containing family, an emerging protein class whose members are involved in key physiological and pathological processes, including cancer, neurodevelopmental and neuropsychiatric disorders, adipogenesis, and genetic diseases [1,2,3,4,5,6,7,8,9,10,11]. The family consists of 25 members whose biological roles, though not yet fully elucidated, are highly diversified. The members of the family are localized to distinct cellular compartments, and their function(s), in contrast to their name, are essentially not linked to potassium channels. Studies carried out in the last two decades have shown that the different members of this protein family play a role in a large variety of often unrelated physiological and pathological processes [12,13,14,15,16,17,18,19,20,21,22,23,24,25]. Interestingly, even single KCTD proteins or sets of closely related homologs are involved in completely distinct processes and diseases. In this context, the case of KCTD1/KCTD15 is paradigmatic, as they are central to embryonic development, cellular homeostasis, and various genetic, metabolic, and oncological disorders [4,5,9,10,11,26,27,28,29,30,31,32,33,34,35,36,37,38,39,40,41,42,43,44,45,46,47]. Although the molecular and structural mechanisms underlying these processes are often obscure, the biochemical role of some KCTDs has been clarified. Specifically, many members of the family are substrate adaptors of the CRL3 ubiquitin ligases (Cullin-RING ligases), effectors of GABA_B2_ receptor (GABA_B2_R) signaling, master regulators of cAMP signaling, and inhibitors of the AP-2 transcription factors [11,19,48,49,50,51,52,53,54,55,56,57,58,59].

**Figure 1:**
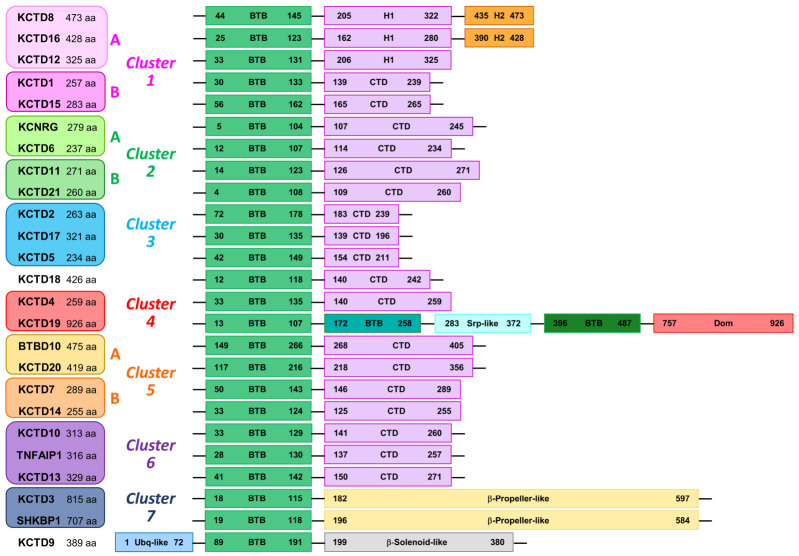
KCTD protein domain organization. The protein members of the family are grouped by using our previous C-terminal domain structure-based clustering [63].

As shown in Figure 1, the molecular organizations of KCTD proteins are somewhat diverse, with the universal presence of the BTB domain, which defines the family. The BTB (Broad-Complex, Tramtrack, and Bric-a-brac also denoted as POZ-Pox virus and Zinc finger—or T1), initially identified in fruit flies [60], is a widespread protein domain [61,62] that generally participates in oligomerization and protein–protein interactions. In addition to the BTB, which is generally located at the N-terminus of these proteins, there is a C-terminal domain (CTD) that presents highly diversified sequences. Indeed, significant sequence similarities are observed for members of the same clade/cluster [63]. A few family members present extra domains (Table 1).

**Figure 2:**
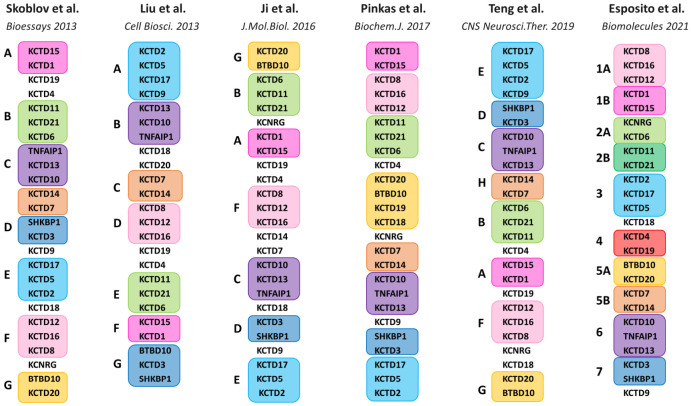
Comparison of the different classification systems proposed for the KCTD protein family in the literature [2,51,54,63,65,66].

Over the years, various clusterings of family members have been proposed (Figure 2). The generally close similarities between the BTB domains [64] and, in contrast, the absence of any widespread sequence similarity in the CTD has made this classification, and, as a consequence, the generation of reliable evolutionary trees, cumbersome.

**Figure 3:**
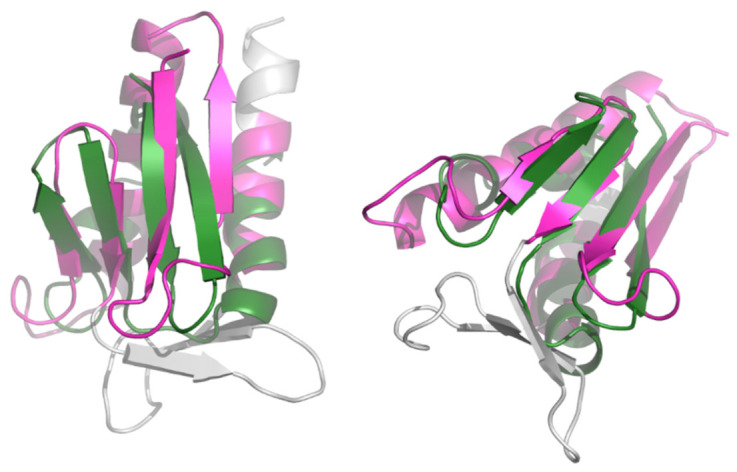
Two views of the structural superposition between the AF-predicted BTB domain of KCTD19 (residues 396–455, forest green) and the corresponding aligned regions of the AF-predicted CTD of KCTD4. In KCTD4, the aligned regions comprise residues 140–196 and 220–229 and are shown in magenta. The segment comprising residues 197–219 and the C-terminal extension (residues 230–235), which are not included in the alignment, are shown in light gray.

The evolutionary and structural trees of the KCTD protein family go from sequence-based paralog trees to novel structural dendrograms based on AlphaFold (AF) predictions. The first foundational classification was proposed by Skoblov et al. (2013) [65], who used the neighbor-joining method to compare both isolated BTB domains and entire amino acid sequences. They proposed a seven-clade structure (groups A–G) that has served as the family’s primary classification (Figure 2). They also showed that the BTB domains of KCTD proteins form a group distinct from the T1 domains of voltage-gated potassium (Kv) channels and included the member BTBD10 in the family. They also attempted to align specific functional modules with these phylogenetic branches. Later that year, Liu et al. (2013) [66] expanded on the evolutionary relationships by mapping known biochemical functions directly onto the tree. They built their paralogue tree based on full amino acid sequences, which mirrored the Skoblov et al. model and maintained the seven-group categorization, but noted that certain members, such as KCTD4, 18, 19, and 20, did not fit perfectly into these groups in their specific analysis (Figure 2). The groups of Privé [54] and Bullock [51] provided a tree that correlated evolutionary position with protein structural properties. They discovered that the multimerization behavior of the BTB domains (see below for details) separates well by evolutionary clade. Later, Teng et al. (2019) [2] refined the sequence-based tree by adding a new clade and expanding the analysis species in addition to humans (mice, zebrafish, Drosophila, C. elegans, and yeast). They proposed the existence of an 8th clade (Clade H) consisting of KCTD7 and KCTD14, and reclassified KCTD9, previously often considered an outlier, within Group E. The most recent tree, produced by Esposito et al. (2021) [63], shifted the focus from sequence similarity to high-confidence structural similarity using AF models. In particular, despite the lack of widespread sequence similarity in the CTDs, they found structural similarities within this domain even among distantly related members of the family. They also detected a strong structural analogy and significant, previously undetected, sequence similarities between the CTDs of KCTD8/12/16 and KCTD1/15, which are all non-binders of cullin 3 (Cul3). These proteins, which were frequently far apart in the other classifications, were grouped in cluster 1 (subclusters 1A and 1B). By grouping KCNRG and KCTD6 with KCTD11 and KCTD21, they suggested that KCNRG belongs to the “KCASH” functional subfamily. Moreover, the absence of a CTD similar to that observed in the other KCTDs in KCTD3, SHKBP1, and KCTD9 led the authors to classify them as “non-canonical” members of the family. Interestingly, they found that one of the BTB domains of KCTD19 structurally resembles the canonical CTD of KCTDs (Figure 3), suggesting a common origin of the major domains of these proteins and representing an evolutionary link between them [63].

Although these classifications share many features and have proven valuable tools for understanding the evolution of these proteins and identifying structure–function correlations, it is important to assess how their differences align with the available structural and functional data. The ability of these classifications, with particular attention to the recent structure-based one [63], to interpret data collected over the last decade will be evaluated in the sections below, which present a chronological journey through the structural biology of KCTD proteins, a topic that has been hitherto reviewed for very specific members of the family [67,68].

## 2. Experimental and Classical Molecular Modeling Structural Studies

Retracing the stages of structural characterization of KCTD proteins reveals the fundamental integration of experimental and theoretical approaches. In this section, the prolific interplay among different structural and computational structural biology techniques is described in detail, generally following the chronological order of the studies.

**Figure 4:**
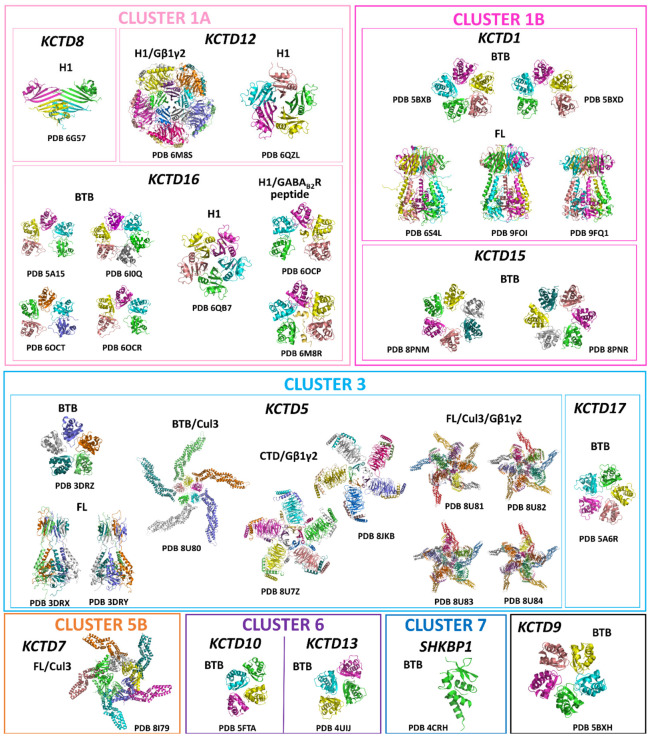
Cartoon representation of the experimentally determined structures of KCTD family members, organized according to their cluster-based classification [63]. For each protein, the corresponding PDB accession code is indicated. Individual chains are shown in different colors to highlight their organization within each assembly.

As of May 2026, the Protein Data Bank (PDB, https://www.rcsb.org/, accessed on 4 May 2026) contains 33 entries related to human KCTD proteins (Table 2 and Figure 4, Figures S1 and S2).

These structures, derived using either crystallographic or cryo-electron microscopy techniques, span a wide range of molecular complexity, from individual domains to large intermolecular complexes. The first 3D structural study of this protein family was the determination of the structure of one of the most extensively investigated KCTDs, KCTD5 [57] (Figure S1 and Figure 4). This seminal study provided some non-trivial clues that were frequently found in other members. Contrary to expectations that KCTD proteins would form tetramers like the potassium (Kv) channels they are named after, the study revealed that KCTD5 assembles into homo-pentamers (five subunits). They also found the full-length protein structure exhibits a “gown-shaped” architecture, with a central cavity spanning two distinct N-terminal (BTB) and C-terminal modules connected by a flexible linker. Indeed, the study found that these two modules are highly dynamic and can swivel by approximately 30° relative to one another. The structural plasticity of the KCTD5 BTB and CTDs was later confirmed by molecular dynamics simulations [69] and shown to be functionally important. In the early stages of KCTD structural biology, molecular modeling was used to elucidate the structural basis of Cul3 recognition.

These predictions were successfully used to identify the hot spot of KCTD11(REN)—Cul3 binding [24,70] and to generate reliable 3D models of these complexes. In particular, it was possible to establish that KCTD5 forms a unique 5:5 hetero-decamer with Cul3, with a peculiar five-pointed pinwheel shape [71]. This complex arrangement was later confirmed by cryo-electron microscopy [54,59] (see below). By discovering that KCTD12 and KCTD15 were unable to bind the cullin, it was also demonstrated that Cul3 anchoring is not a universal feature of KCTD proteins, with selectivity determined by specific structural loops [49]. The elucidation of the KCTD-Cul3 recognition modes also led to the identification of Cul3-based peptides that could bind with good affinity to KCTD5 and KCTD11 [72,73]. The modeling with Cul3 was also successfully extended to Insomniac (INC), the Drosophila melanogaster (fruit fly) ortholog of the human KCTD5/2/17 cluster, which plays a crucial role in sleep homeostasis [74]. The validation of this INC-Cul3 complex by SAXS data represented the first structural characterization of this assembly type and highlighted its highly dynamic nature [74].

Subsequent studies were focused on isolated BTB domains and unraveled their highly versatile oligomerization (Figure 4). The structural characterization of the KCTD9 BTB domain [54] confirmed the propensity of these domains to adopt pentameric states. Surprisingly, the equivalent domain of KCTD1 could form both closed and open (C-shaped) pentameric rings. New and variegated oligomeric states emerged from the crystallographic study by Pinkas et al. on the BTB domains of SHKBP1 (monomer), KCTD16 (open pentamer), KCTD17 (closed pentamer), and KCTD10 and KCTD13, which both formed two-fold symmetric tetramers [51]. Interestingly, except for KCTD16, which does not bind Cul3, in all other cases, the presence of Cul3 can induce their reassembly into 5:5 hetero-decamers. The tendency of BTB domains to preferentially adopt either closed or pentameric states was also corroborated by a negative staining electron microscopy study [75]. The versatility of these BTBs was thoroughly investigated using molecular dynamics simulations, which indicated that the monomeric states of these domains are generally stable and rigid [76]. The simulations also revealed that the open pentameric states are more stable in proteins (e.g., KCTD12 and KCTD16) in which the gap between subunits is essential for anchoring their biological partner, the GABA_B2_R receptor (see below for further details). The structural versatility of these BTB domains has been recently further corroborated by crystallographic studies in which the formation of non-regular [52] or regular hexameric states with C6 symmetry has been observed [11].

Mutations of KCTD proteins are involved in several genetic diseases. Among them, KCTD1 variants associated with the scalp-ear-nipple syndrome (SENS) have been widely investigated, also from a structural point of view. These mutations, identified by Marneros et al. [9], cause this rare, autosomal-dominant disorder characterized by cutis aplasia of the scalp, minor anomalies of the external ears, digits, and nails, and are located, with the exception of P20S, in the BTB domain. The biophysical characterization of some SENS-related representative mutants (P20S, H33P, G62D, D69E, and H74P) indicates that all of these mutations resulted in a significant destabilization of the KCTD1 structure [44]. Except for P20S, the analysis of the BTB domain of KCTD1 provided a rationale for the observed destabilization. Interestingly, these KCTD1 variants were found to undergo amyloid-like aggregation. As a consequence, all examined SENS-causing proteins are unable to interact with their biological partner, the transcription factor AP-2α, as also found in a more recent study [35]. The propensity of the mutants to form aggregates provides a molecular explanation for the autosomal-dominant nature of SENS, since these aggregates can act as templates, triggering the aggregation of wild-type KCTD1 proteins in heterozygous patients and sequestering them in an inactive state. More recent studies have shown that these variants tend to form aggregates also in cells [10]. One open issue was the observation that the P20S mutation, located in a presumed unstructured region of the protein, could destabilize the protein. This puzzling observation has recently been resolved by two independent studies [55,56], which report the crystallographic structures of KCTD1 and its P20S variant. These studies demonstrated that KCTD1 has an intricate architecture that relies on 3D domain swapping, with the CTD forming a central channel in the pentamer that can sequester potassium, sodium, phosphate, and iodide ions. Interestingly, the pre-BTB region harboring the P20S mutation adopts a polyproline conformation and actively participates in the swapping.

In a recent study, Miller et al. linked KCTD15 mutations to a distinctive dysplasia [11]. The authors found two missense mutations, both acting via a dominant-negative mechanism to disrupt the native pentamer. Interestingly, while the mutation D104H resulted in the KCTD15 partial unfolding and monomerization, G88D mutation was found to induce a closed hexameric assembly (Figure 4). This unexpected finding provided further support to the oligomerization versatility of the BTB domains of KCTDs. Later prediction analysis showed that AlphaFold was able to capture this hexameric state [68].

In the last decade, experimental studies have provided important insights into the structural basis of partner recognition by KCTDs. The major studies provided the structural basis for how KCTDs regulate neurotransmitter signaling via GABA_B2_R receptors [50,53]. In two independent studies, it was shown that the BTB domain of KCTD16 forms an open pentamer in which the GABA_B2_R C-terminal peptide is tightly bound. In addition, Kruse et al. determined the structure of the KCTD12 H1 domain in complex with Gβ1γ2, with a 5:5 stoichiometry (Table 2 and Figure 4). They found that the H1 domain adopts a β-propeller fold and that the BTB and H1 domains in the full-length protein are physically separated by ~35 Å. This architecture allows the H1 domain to “strip” Gβ1γ2 subunits from GIRK channels, desensitize signaling, and target KCTD16 and G-protein binding. Intriguingly, in addition to regulating Gβ1γ2 activity, KCTDs also play a crucial role in their processing. Indeed, KCTD5 is responsible for the ubiquitination of these proteins, acting as a substrate adaptor. The determination of the structure of the 5:5 complex formed by KCTD5 and Gβ1γ2 by cryo-electron microscopy unravels that each CTD of KCTD5 interacts with blade 1 of the torus of Gβ1γ2 [58] (Figure 4). This binding site overlaps the region where Gβ1γ2 subunits normally bind, explaining why KCTD5 targets only free Gβ1γ2 released from the heterotrimer. Notably, unlike the KCTD12 complex, in which the subunits are tightly packed and interact, the subunits in the KCTD5 complex are spatially isolated. In the same study, Jiang et al. [58] also reported the structure of the KCTD7–Cul3 complex. This structure provided the first high-resolution map of the N-terminal domain (NTD) of Cul3 bound to a pentameric KCTD protein. In line with predictions reported for other KCTDs [49,71,74], the 5:5 complex presents a five-pointed pinwheel shape, with two adjacent BTB subunits of the KCTD7 pentamer contributing to the anchoring of the cullin. This observation further corroborates the proposal that the KCTD-based E3 ligase may enable the simultaneous ubiquitination of five subunits. This study also mapped over 40 mutations associated with Progressive Myoclonic Epilepsy (PME) onto this KCTD7 structure, grouping them into distinct (dis)functional categories, including folding, oligomerization, Cul3-binding, and substrate-binding disruption.

The structural basis of the KCTD5 impact on G-protein signaling has also been investigated through the cryo-electron microscopy characterization of the 5:5:5 complex formed by KCTD5/Cul3/Gβ1γ2 [59]. The most striking finding of this work was the discovery of a massive dynamic of the complex centered on a specific 10-residue hinge region of the protein. Deleting seven residues from this “waist” significantly impairs ubiquitination activity, proving that this flexibility is a mechanical requirement for the reaction to proceed. As other reports have shown, these data indicate that oligomerization of a substrate receptor can generate a polyvalent E3 ligase complex and that the internal dynamics of the substrate receptor can position a structured target for ubiquitylation within a CRL3 complex.

## 3. Machine-Learning-Based Predictive Approaches

As illustrated in the previous section, the experimental characterization of KCTDs, even though it has been efficiently integrated with classical homology modeling, has followed a tortuous path and has yielded only a very partial description, though insightful for the investigated systems, of the structural properties of KCTD proteins. Indeed, as reported in Table 2, of the twenty-five multidomain members of the family, each involved in multiple functional partnerships, 3D models are available for only three KCTDs (KCTD1, KCTD5, and KCTD7), and for some other members, only a domain has been characterized, either the BTB (KCTD9, KCTD10, KCTD13, KCTD15, KCTD16, KCTD17, and SHKBP1) or the CTD (KCTD8, KCTD12, and KCTD16) (Figure S2). Regarding the partnerships of the BTB domain, the atomic-level interactions with Cul3 have been experimentally characterized for KCTD5 and KCTD7 [58], whereas the structure of the complex formed by this domain with the C-terminal peptide of GABA_B2_R has been reported for KCTD16 [50,53]. Finally, the only complexes involving the CTDs are those formed by KCTD16 and KCTD5 with the Gβ1γ2 subunits of GIRK channels.

As in many other cases [64,77,78,79], the advent of machine-learning-based predictive approaches, such as AlphaFold [80], has completely changed the landscape. Indeed, investigations carried out over the last lustrum have provided comprehensive, family-wide data on several knowledge gaps in the structural biology of KCTD proteins. The specific topics covered in these analyses have followed the evolution of AlphaFold developments over recent years. The first application of this approach on KCTD proteins was conducted using the structures of protein single chains that were deposited in the AlphaFold Protein Structure Database—a joint partnership between Google DeepMind and EMBL-EBI (European Bioinformatics Institute) (https://alphafold.ebi.ac.uk/, accessed on 1 November 2021), which was officially launched on 22 July 2021. Using this structural information, available only at the single-chain level, we provided a global structural view of the KCTD protein family, identifying critical relationships previously obscured by a lack of sequence similarity [63]. Surprisingly, AF models revealed that these domains share a common fold, which is typically characterized by a single β-sheet surrounded by two to five α-helices. Using the newly identified KCTD-CTD as a reference, the authors generated a novel structural dendrogram (pseudo-phylogenetic tree) that reclassifies the family into seven distinct clusters (Figure 2) [63]. These clusters comprise 22 of the 25 family members; the only exceptions are KCTD3, SHKBP1, and KCTD9, which lack a CTD-like domain and are therefore identified as non-canonical KCTDs. Intriguingly, the comparison of the structure of the KCTDs shows that the third BTB domain (BTB3) of KCTD19 exhibits significant structural analogies with the CTDs of the KCTD protein family (Figure 3). This suggests that the BTB3 domain of KCTD19 may link the canonical BTB fold to the KCTD-CTD structure, leading to speculation that the BTB and CTD might share a common ancestor, potentially evolving from the same original module.

**Figure 5:**
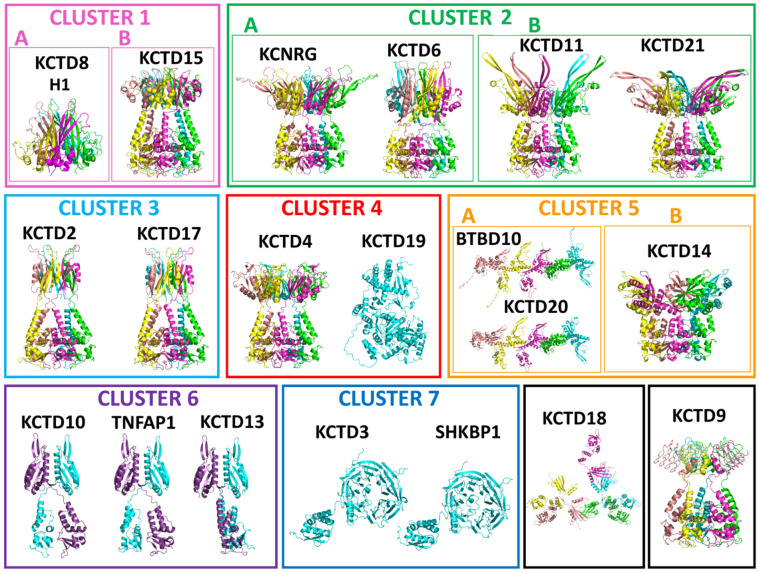
Cartoon representation of the AF-predicted structures of KCTD family members for which a full experimental structure is missing. Individual chains are depicted in different colors. Predictions of pentameric states failed for KCTD18 and for proteins of clusters 5A and 6 (the results are shown). AF predictions for proteins of cluster 6 indicate a putative dimeric assembly. Predictions of monomeric states are reported for KCTD19, KCTD3, and SHKBP1 (long disordered stretches have been omitted).

The release of AlphaFold-multimer [81,82], which enabled prediction of the structures of both homo- and hetero-oligomers, led to significant progress in elucidating the structural features of KCTDs. Indeed, this tool was successfully used to systematically predict the (presumed) functional oligomeric states of the entire KCTD protein family, providing a global structural view of previously uncharacterized assemblies [79] (Figure 5), including those for which no structural information, even of fragments or domains, was available.

**Figure 6:**
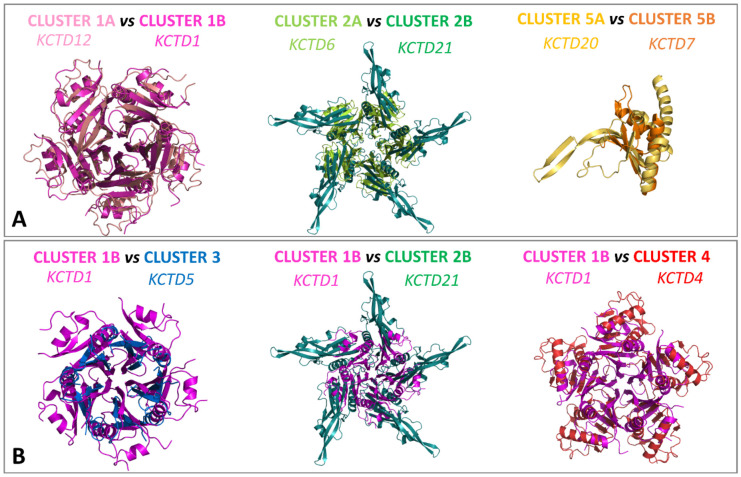
Structural alignment of the CTDs of selected KCTD family members within the same cluster but different subclusters (**A**), and comparison of KCTD1 (magenta) with KCTD5 (blue), KCTD21 (green), and KCTD4 (red) (**B**). The selected members are specified in the figure. Experimentally determined structures were used for KCTD1 (PDB ID: 6S4L), KCTD5 (PDB ID: 3DRX), and KCTD7 (PDB ID: 8I79), whereas three-dimensional models of the KCTD4, KCTD6, KCTD12, and KCTD21 pentamers were retrieved from our in-house database of KCTD predicted structures (https://alphafold.ibb.cnr.it/, accessed on 1 March 2026). For KCTD20, the corresponding EBI AlphaFold model was used. Structural superpositions were performed using a combination of US-align and PyMOL (version 3.1).

The BTB domains of these proteins, consistent with experimental data, in most cases assume similar, canonical pentameric structures. On the other hand, the oligomerization of the CTDs led to a variety of different three-dimensional structures. Indeed, despite the general lack of sequence similarity across different clusters, the CTDs of most KCTDs (clusters 1–4 of Figure 1) share a common structural theme characterized by a multimeric propeller-like fold in which the five CTD assembly forms a central cavity delimited by five exposed and regular β-strands (Figure 5). The preservation of this fold is notable because these CTDs vary significantly in size, ranging from approximately 60 to 120 residues. As a result, the structures of some CTDs from different clusters could be successfully superimposed (Figure 6).

The predictions also highlighted a unique architecture of cluster 5B (KCTD7 and KCTD14) whose members form a circle-like CTD assembly with a central cavity that is significantly larger than those found in clusters 1–4 (Figure 5). The correctness of this prediction has been assessed by the cryo-electron microscopy determination of the structure of KCTD7 [58], which was in close agreement with the one previously predicted (Figure S3) [79]. A puzzling yet unresolved issue is that the members of cluster 6 (KCTD10, KCTD13, and TNFAIP1) exhibit a strong tendency to form stable dimers rather than closed pentamers in full-length proteins, despite the propensity of their BTB domains to adopt pentameric states [51]. In some cases (KCTD18, KCTD20, and BTBD10), all attempted predictions for oligomeric states were unstable or unreliable, suggesting they may operate in different, currently uncharacterized architectures.

The possibility of predicting multimeric protein structures offered the opportunity to investigate, again in a family-wide mode, the interactions of these proteins with Cul3, one of the most important biological partners [64]. Although AlphaFold has some well-known limitations in predicting the structural impact of limited sequence variations [81,83], it displayed an impressive ability to discriminate between family members that interact with Cul3 and those that do not. Indeed, reliable and stable models were predicted for all 15 known binders in the family, regardless of whether they were monomeric or pentameric states, whereas for the others, the algorithm predicted only unstable complexes with high expected errors. Based on these predictions, it was possible to assess, as a general trend, that KCTDs-Cul3 partnerships rely on two distinct spots. This primary binding site involves the α2β3 loop and the α4α5 helical hairpin of the KCTD BTB domain, while a second adjacent KCTD subunit contributes to the binding via its α1 helix, which interacts with the H1 and H2 helices of Cul3 [64].

In addition to these comprehensive studies, AlphaFold has been used in specific cases, such as modeling the KCTD4-CLIC1 protein [84] and in the recent seminal study that identified KCTD10 as a sensor for co-directional transcription–replication conflicts [12].

## 4. Hetero-Oligomers Formed by KCTD

The structural complexity, oligomeric organization, and significant sequence-structure similarity enable the formation of hetero-oligomers within the KCTD family. These have been detected in several contexts and frequently shown to play a significant functional role. The survey of the current literature indicates that hetero-complexes may form not only within the same subcluster or cluster but also between members of different clusters, even non-contiguous ones. In the following paragraphs, the identified hetero-oligomers are organized by increasing sequence/molecular similarity among the KCTD partners involved.

Considering the sequence/structure similarity of the KCTD belonging to the same subcluster/cluster, it is not surprising that the formation of hetero-oligomers between these sub-ensembles has been frequently observed. Particularly interesting, given its important functional implications, are the hetero-oligomers formed by KCTD12 and KCTD16 in cluster 1A [85]. In this study, the properties of the KCTD12/KCTD16 hetero-oligomers were shown to be quite different from the KCTD12 or KCTD16 homo-oligomers, thus demonstrating that simultaneous assembly of distinct KCTDs at the receptor increases the molecular and functional repertoire of native GABA_B2_R receptors and modulates physiologically induced K+-current responses in the hippocampus. Remarkable results in this context have been recently achieved for KCTD1 and KCTD15 (cluster 1B). Initial indications of the interactions between the two proteins emerged from interactome analysis [5]. Indeed, upon demonstrating that KCTD15 is critical for cardiac outflow tract development, whereas KCTD1 regulates distal nephron function, it has been found that KCTD1 and KCTD15 can form multimeric complexes and compensate for each other’s loss [10]. Interestingly, disease mutations in one of these proteins are dominant-negative, resulting in a lack of both KCTD1 and KCTD15 functions. These results have been interpreted by assuming that the loss of function is likely due to the propensity of these proteins to form amyloid-like aggregates, and that the increased propensity to aggregate of one protein associated with the mutation serves as a template for the aggregation of the other protein’s wild-type form [10], consistent with a previous hypothesis [44].

Within cluster 2, KCTD6, KCTD11, and KCTD21, which are collectively denoted as KCASH (KCTD-Containing Cullin3 Adaptors, Suppressors of Hedgehog), can form highly specific hetero-oligomeric complexes, though they do not all interact interchangeably [86]. Indeed, although KCTD11 interacts with both KCTD6 and KCTD21, these two latter proteins do not form hetero-oligomers. The formation of these hetero-complexes enables KCTD6, which is unable to bind HDAC1 as a homo-oligomer, to participate in HDAC1 ubiquitination.

It has been shown that the KCTD2-KCTD5 hetero-oligomer (cluster 3) associates with Cul3 through the KCTD5 subunit and recruits Gβ1γ2 through both KCTD proteins in response to G-protein activation [87]. The in vitro hetero-association of KCTD5 with KCTD2 and KCTD17 has recently been reported and securely assessed [88]. These findings are fully consistent with the observation that these KCTD proteins have redundant functions in controlling cellular growth [89].

The PDIP1 KCTD sub-family (KCTD10, KCTD13, and TNFAIP1), which constitutes cluster 6, also forms functional hetero-complexes. The physical and functional interaction between KCTD10 and TNFAIP1 was first comprehensively described in 2012 by Hu et al. [90], who showed that it promotes the degradation of KCTD10 and inhibits the transcriptional activities of NF-κB and AP-1. Very recently, it has been shown that KCTD10 acts as a critical negative regulator of KCTD13. Indeed, by forming a hetero-complex, KCTD10 recruits the Cul3 ubiquitin ligase machinery to target KCTD13 for proteasomal degradation [17].

In addition to experimental evidence of cross-interactions among members of the same cluster, literature studies have also shown that inter-cluster hetero-associations occur. These have been detected mainly by members of clusters that are contiguous in the structure-based classifications [63]. In particular, in their analysis of the broad hetero-oligomerization network of KCTD5, Muntean and coworkers [88] discovered that the protein interacts with a dozen other KCTDs. Although most of these interactions are mediated by the BTB domain of these KCTDs, they also found that in some cases the contribution of the CTD was important. This is particularly relevant for the hetero-associations established by KCTD5 with the members of cluster 1A. Both KCTD5 domains are required for the recognition of the full-length KCTD16. Moreover, they found that the KCTD5-KCTD8 association is mediated exclusively by the KCTD5 C-terminus and that full-length KCTD12 interacts with the isolated KCTD5 C-terminus. Collectively, these findings suggest favorable interactions of the CTDs of these proteins. Other important intercluster hetero-oligomerizations have been reported by De Smaele and coworkers, who showed that members of cluster 2 can interact with those of cluster 1B [86]. In particular, it was initially shown that KCTD15 forms hetero-oligomers specifically with KCTD21 but does not interact with KCTD11 or KCTD6 [41]. More recent investigations have shown that KCTD1 can form hetero-oligomers with both KCTD11 and KCTD21 [38]. Interestingly, they found that the formation of these hetero-oligomers stabilizes KCTD11 and KCTD21, protecting them from proteasomal degradation and thereby enhancing their ability to suppress the Hedgehog pathway. Finally, as an extension of these findings, the hetero-complex formation has also been observed for KCTD and KCTD-like proteins isolated from different species. Indeed, it has been shown that mammalian members of the KCTD cluster 3 can hetero-multimerize with the Drosophila protein Insomniac [91].

**Figure 7:**
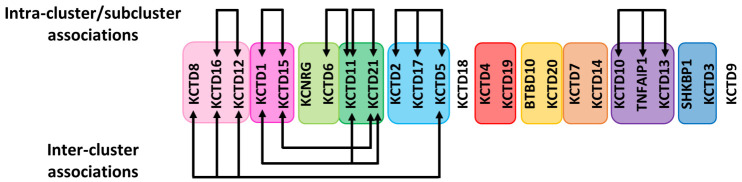
Schematic overview of the hetero-partnerships among KCTD family members with known functional implications. KCTD proteins for which experimental evidence supports the formation of hetero-oligomeric assemblies are connected by arrows. Interactions between members belonging to the same cluster or subcluster are shown in the upper part of the figure, whereas interactions involving members from different clusters are reported in the lower part.

These experimental findings have been collectively summarized in the scheme reported in Figure 7.

**Figure 8:**
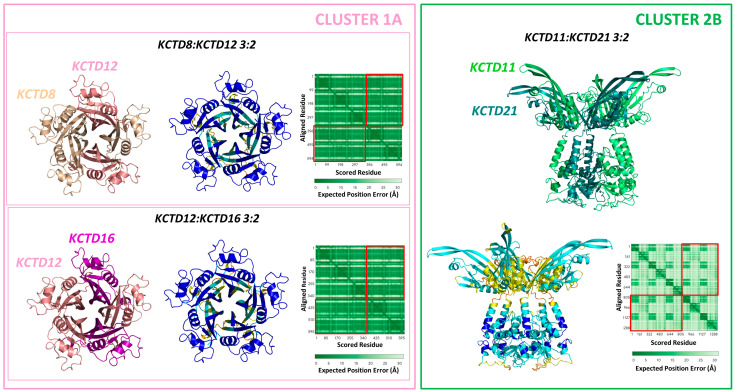
Cartoon representations of stable AF-predicted KCTD assemblies obtained by combining chains from two different KCTD family members within the same subcluster. The models illustrate the structural arrangement of the resulting pentameric assemblies, with chains colored according either to their protein of origin or to the AF per-residue confidence metric (pLDDT) as follows: blue for pLDDT > 90, cyan for 70 < pLDDT ≤ 90, yellow for 50 < pLDDT ≤ 70, and orange for pLDDT < 50. Predicted Alignment Error (PAE) matrices are also shown; regions highlighted in red boxes indicate inter-chain interfaces between the different KCTD members. For members of cluster 1A, only the H1 domain is considered.

**Figure 9:**
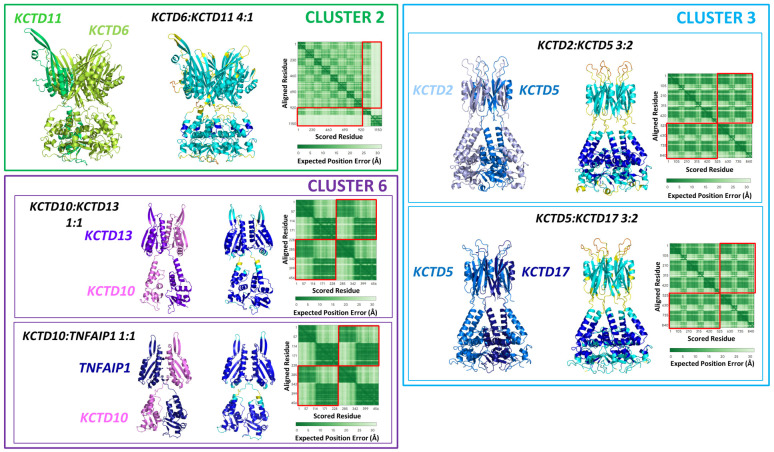
Cartoon representations of stable AF-predicted KCTD assemblies obtained by combining chains from two different KCTD family members within the same cluster. The models illustrate the structural arrangement of the resulting assemblies, with chains colored according either to their protein of origin or to the AF per-residue confidence metric (pLDDT) as follows: blue for pLDDT > 90, cyan for 70 < pLDDT ≤ 90, yellow for 50 < pLDDT ≤ 70, and orange for pLDDT < 50. Predicted Alignment Error (PAE) matrices are also shown; regions highlighted in red boxes indicate inter-chain interfaces between the different KCTD members.

**Figure 10:**
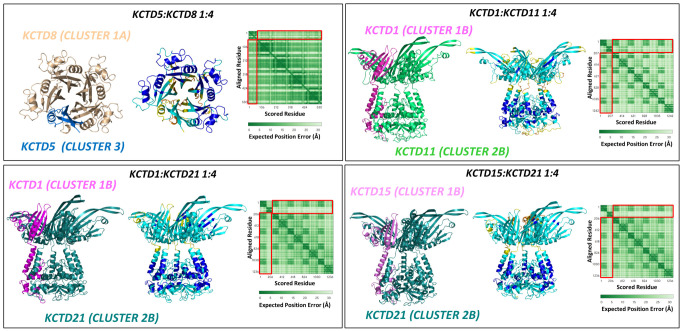
Cartoon representations of stable AF-predicted KCTD assemblies obtained by combining chains from two different KCTD family members within different clusters. The models illustrate the structural arrangement of the resulting pentameric assemblies, with chains colored according either to their protein of origin or to the AF per-residue confidence metric (pLDDT) as follows: blue for pLDDT > 90, cyan for 70 < pLDDT ≤ 90, yellow for 50 < pLDDT ≤ 70, and orange for pLDDT < 50. Predicted Alignment Error (PAE) matrices are also shown; regions highlighted in red boxes indicate inter-chain interfaces between the different KCTD members.

Unfortunately, no experimental structural information is available for hetero-oligomers, which could help interpret the role of hetero-oligomerization in KCTD activity modulation. This is not surprising as it would be difficult to obtain a pure, homogeneous sample with a single complex stoichiometry. So far, AF predictions for the hetero-association of KCTD1/KCTD15 have been reported [10]. Interestingly, these investigations show a tendency for these proteins to form hetero-pentamers rather than homo-pentamers, thereby confirming their close association and interchangeability. Stimulated by these observations, we here explored the possibility of predicting other hetero-complexes formed by KCTD proteins, as experimentally validated. Present predictions were made using the AlphaFold3 (AF3) algorithm (https://alphafoldserver.com/, accessed on 1 March 2026) [92] using default settings (see Supplementary Material for details). The best-predicted model (model 0) out of the five provided by AF3 was considered. The reliability of these predictions was evaluated by analyzing the Predicted Aligned Error (PAE) matrices (Figure S4) and the per-residue Local Distance Difference Test (pLDDT), whose values were used as a color code in the prediction figures (Figure 8, Figure 9, Figure 10, Figures S5 and S6). The assessment of the reliability of the hetero-complexes was performed by comparing the PAE regions corresponding to the homomeric interactions with the heteromeric ones. The similarity among these regions was considered evidence of the reliability of the hetero-complexes. We initially assessed the ability of proteins within the same subcluster to form hetero-complexes. As expected from global similarity, these trials yield positive results. As shown in Figure 8 and Figure S5, which report assemblies with a 3:2 stoichiometry, the CTDs of KCTD8, KCTD12, and KCTD16 undoubtedly assemble with low expected errors in their intermolecular interactions, as deduced from the PAE matrices. Similar results are obtained for the full-length KCTD11/KCTD21, although in this case the predicted errors are slightly larger (Figure 8).

When we considered the complex formed by the full-length KCTD6/KCTD11, which belong to the same cluster but distinct subclusters, the only model showing some reliability is the 4:1 one (Figure 9, Figures S5 and S6), whose formation is driven by the BTB domains and the presence of 4 KCTD6 CTDs. This is not surprising considering the complete sequence diversity of the CTDs. In any case, the prediction shows that the presence of a single KCTD11 CTD is tolerated. This is a general trend for the mixing of highly diverse CTDs. Very low expected errors are obtained for the hetero-pentameric complexes of KCTD2/KCTD5/KCTD17 and for the dimeric hetero-oligomers of KCTD10/KCTD13/TNFAIP1 (Figure 9 and Figure S5).

We then evaluated the predictions obtained for hetero-complexes formed by KCTDs from different clusters. Stable complexes are obtained by mixing the KCTD5-KCTD8 CTDs with a 1:4 stoichiometry (Figure 10). Still reliable, but with larger expected errors, are the predictions with other stoichiometries, except for the 2:3 complex (Figure S6), for which no reliable complex is obtained. These findings suggest that the most stable complexes are obtained when the larger CTD is prevalent in the stoichiometry. A similar pattern is observed for KCTD5-KCTD16 hetero-complex formation (Figure S5).

The predictions also show that KCTD1 and KCTD15 (cluster 1B) can form stable pentamers with KCTD21 (cluster 2B) (Figure 10 and Figure S5). A stable pentamer is also observed for KCTD1/KCTD11 association (Figure 10).

In conclusion, the literature analysis of KCTD hetero-complex formation suggests that, most likely, mixing corresponds to clusters that are close in the structure-based organization of these proteins [63]. Although the derived models require experimental validation, present predictive analyses provide some structural information that could be useful for the interpretation of future experimental data.

## 5. Concluding Remarks and Perspectives

The characterization of protein function/structure relationships, despite the enormous successes achieved over the years of intense study that have modeled the structural biology revolution, remains a complex task that often requires substantial economic and human resources. Although the experimental methodologies used to characterize them have advanced tremendously in the last few decades, applying these approaches is time-consuming and provides no a priori guarantee of success. Fortunately, the possibility of obtaining a reliable three-dimensional model via protein structure prediction based solely on sequence information and using machine-learning approaches is rapidly changing the scenario. The application of these tools also allows structural biologists to move beyond the reductionist approach of studying proteins one by one, enabling the simultaneous prediction and comparison of the structures of all members of protein families.

The analysis of the state of the art in the structural characterization of KCTD proteins, reported here, is prototypical in this context. Although a large body of data has highlighted the crucial role of these proteins in several physiological and pathological processes, their structural characterization has proven to be a complex endeavor. Indeed, after two decades of attempts to determine KCTD three-dimensional structures, the experimental information collected, although crucial to understanding the functional properties of individual members, has remained limited at the family level. Indeed, as illustrated in the previous section, the structure of only a few family members is known (Table 2), and limited information about their partnerships is available. Nevertheless, these studies, as detailed above, often complemented with classical homology modeling and biophysical characterization, have provided information on the global architecture of some members of the family (KCTD1, KCTD5, and KCTD7), the structural basis of some important functional partnerships (Cul3, GABA_B2_R, Gβ1γ2), and on the structural mechanism underlying genetic diseases. Even the structural characterization of isolated domains, such as the BTBs, has been informative. Indeed, although the observed versatility of these domains in adopting oligomeric states is, in part, due to protein truncation, it may also have functional implications that warrant attention. In general, the stability of these domains, independent of their oligomeric organization, enables these proteins to undergo structural rearrangements that could be exploited in specific functional contexts. A corollary of these considerations is that the assumption that the BTB domains drive oligomerization of these proteins must be significantly revised, also considering that, in some cases, CTD association is more stable than BTB one [5,75]. It is likely that BTB oligomerization and its versatility are important in creating the optimal surface for intermolecular partnerships. This is evident from the open pentameric structure required to bind the GABA_B2_R C-terminal peptide, or from the involvement of two subunits of the closed pentamer in anchoring Cul3. Further studies are required to see whether this scheme applies to the recognition mechanism, currently unknown at the atomic level, of the third main partner of these BTB domains, the AP-2 transcription factors.

The advent of the AlphaFold era significantly increased the structural information available for these proteins. Although it should be kept in mind that these sequence-derived three-dimensional models require some experimental validation, they have significantly expanded our knowledge, essentially by enabling family-wide studies. Particularly relevant has been the finding that the CTDs of these proteins, although often lacking sequence similarity, are structurally related. This has contributed to the development of a new structure-based classification of these proteins that could help provide a global view of how structure–function relationships are modulated within the family [63]. Indeed, the grouping of KCTD proteins based on sequence comparison is not trivial. Their two common domains (BTB and CTD) exhibit sequence similarity that falls at the two extremes, making their classification difficult: BTB sequences are generally highly (too) similar, and CTD sequences are frequently unrelated. The structure-based KCTD grouping (Figure 2) explains well the similarity of the KCTD oligomerizations obtained from predictive studies.

As detailed in the previous section, this classification is also consistent with the observed propensity of these proteins to cross-associate, forming hetero-complexes made of different members of the family. The high-oligomeric states of these proteins, which generally form pentamers, allow them to operate by simultaneously binding multiple partners and to form mixed oligomers, whose functional importance has been demonstrated. Notably, the formation of these hetero-oligomers has been observed between members that are in the same or in contiguous clusters of the structure-based classification (Figure 2). AF predictions, here reported, provide plausible three-dimensional models of these hetero-complexes. Importantly, the similarity of the structure and not of the function dictates the mixing, as, for example, cullin-binding and cullin-non-binding KCTDs form mixed assemblies. This suggests that multiple functions may be associated with these mixed forms. A single hetero-complex may integrate the distinct functions of the two KCTDs involved in its formation, thereby yielding new functionalities.

The contribution of machine-learning-based approaches has been crucial in filling some gaps in the structural knowledge of these proteins; however, many open issues warrant attention. Although this approach has been used and important data on KCTD partnerships have been collected [12,64], AF contribution to determining the basis of partner recognition by KCTDs remains limited. Post-translational modifications of KCTDs have only been sporadically reported [93]. Nevertheless, it is important to note that substrates often undergo such modifications to enable their recruitment by E3 ligases [94]. It is possible that some of these interactions are mediated by post-translational modifications whose handling by these methodologies is extremely poor. Since the definition of these interactions is essential for developing new compounds that modulate them, as already done in a limited number of cases [73,95], further efforts using all the available repertoire of structural biology experimental and computational techniques are eagerly needed.

## Figures and Tables

**Table 1:** Domain organization of KCTD family members containing additional domains beyond the canonical BTB and CTD regions. Monomeric models retrieved from the EBI AlphaFold Protein Structure Database are also shown, with domains colored differently to highlight their structural organization.

Cluster	Protein	UniProtKB	Domains (Residue Range)	Predicted Model
1A	KCTD8	Q6ZWB6	**BTB** (44–145) **H1** (205–322) **H2** (435–473)	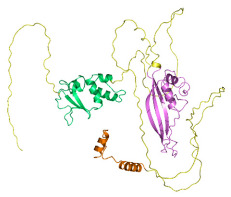
	KCTD12	Q96CX2	**BTB **(33–131) **H1** (206–325)	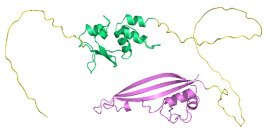
	KCTD16	Q68DU8	**BTB** (25–123) **H1** (162–280) **H2 **(390–428)	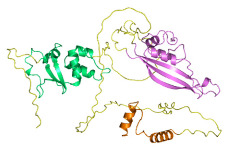
4	KCTD19	Q17RG1	**BTB1** (13–107) **BTB2** (172–258) **Srp-like** (283–372) **BTB3** (396–487) **DOM** (757–926)	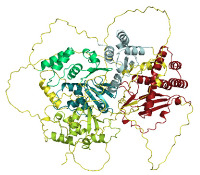
7	KCTD3	Q9Y597	**BTB **(18–115) **β-Propeller-like** (182–597)	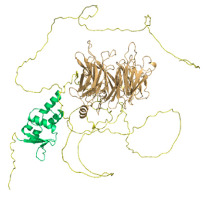
	SHKBP1	Q8TBC3	**BTB **(19–118) **β-Propeller-like **(196–584)	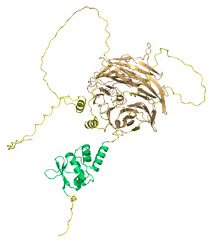
-	KCTD9	Q7L273	**Ubq-like** (1–72) **BTB **(89–191) **β-Solenoid-like** (199–380)	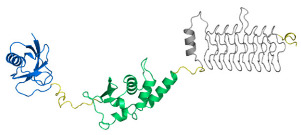

**Table 2:** Structures of KCTD proteins in the PDB.

Cluster	Protein	Domain/Complex	PDB Entry	Released	Resolution (Å)	KCTD Residue Range	Oligomeric State	Reference
1A	KCTD8	H1	6G57	2019	2.80	201–322	Tetramer	Pinkas et al. To be published.
	KCTD12	H1	6QZL	2019	1.98	202–325	Pentamer	
		H1/Gβ1γ2	6M8S	2019	3.71	200–325	Pentamer (H1) Pentadecamer (complex)	[50]
	KCTD16	BTB	5A15	2015	2.76	16–133	Open pentamer	[51]
			6I0Q	2019	2.30	22–124	Hexamer	[52]
			6OCT	2019	2.80	22–134	Open pentamer	[53]
			6OCR	2019	2.28	22–134	Open pentamer	
		BTB/ GABA_B2_R peptide	6OCP	2019	2.35	22–134	Open pentamer (BTB) Octadecamer (complex)	
		BTB/ GABA_B2_R peptide	6M8R	2019	3.20	23–124	Open pentamer (BTB) Hexameric (complex)	[50]
		H1	6QB7	2019	2.23	126–286	Pentamer	Pinkas et al. To be published.
1B	KCTD1	BTB	5BXB	2015	2.17	29–132	Pentamer	[54]
			5BXD	2015	1.80	29–132	Open pentamer	
		FL	6S4L	2020	2.42	28–257	Pentamer	[55]
			9FOI	2025	2.71	1–257	Pentamer	
			9FQ1	2025	2.60	1–257	Pentamer	[56]
	KCTD15	BTB	8PNM	2024	1.94	52–165	Hexamer	[11]
			8PNR	2024	2.25	51–165	Hexamer	
3	KCTD5	BTB	3DRZ	2009	1.90	40–145	Pentamer	[57]
		FL	3DRX	2009	3.11	34–234	Pentamer	
			3DRY	2009	3.30	34–234	Pentamer	
		CTD/ Gβ1γ2	8JKB	2023	3.27	153–211	Pentamer (CTD) Pentadecamer (complex)	[58]
			8U7Z	2023	2.97	152–234	Pentamer (CTD) Pentadecamer (complex)	[59]
		BTB/ Cul3(NTD)	8U80	2023	3.60	40–153	Pentamer (BTB) Decamer (complex)	
		FL/Cul3/ Gβ1γ2	8U81	2023	3.82	40–234	Pentamer (CTD) 20-mer (complex)	
			8U82	2023	3.84	40–234	Pentamer (CTD) 20-mer (complex)	
			8U83	2023	3.98	40–234	Pentamer (CTD) 20-mer (complex)	
			8U84	2023	3.88	40–234	Pentamer (CTD) 20-mer (complex)	
	KCTD17	BTB	5A6R	2015	2.85	13–124	Pentamer	[51]
5B	KCTD7	FL/Cul3	8I79	2023	2.80	1–289	Pentamer (KCTD) Decamer (complex)	[58]
6	KCTD10	BTB	5FTA	2016	2.64	26–135	Tetramer	[51]
	KCTD13	BTB	4UIJ	2015	2.70	27–144	Tetramer	
7	SHKBP1	BTB	4CRH	2014	1.72	18–120	Monomer	
-	KCTD9	BTB	5BXH	2015	2.76	89–191	Pentamer	[54]

## Data Availability

The coordinates of the AF3-predicted models here described are available on request from the authors and at https://alphafold.ibb.cnr.it/ (accessed on 1 March 2026).

## Supplementary Materials

The following supporting information can be downloaded at: https://www.mdpi.com/article/10.3390/ijms27135745/s1.
