# Supplementary material for: A Global Analysis of the Complex Structural Organization of KCTD Proteins and Their Functional Implications
Source: Int J Mol Sci. 2026 Jun 25;27(13):5745. doi: 10.3390/ijms27135745 (PMC13361646; doi:10.3390/ijms27135745)
Supplement: Supplementary file 1 [file ijms-27-05745-s001.zip › ijms-4393607-supplementary.pdf]

Supplementary Material

AlphaFold3 (AF3) predictions

AF3 predictions were generated using the AlphaFold Server (https://alphafoldserver.com/, accessed on 1 March 2026) with default settings. For each prediction, the top-ranked model (model 0) among the five models generated by AF3 was selected for subsequent analyses. Model reliability was assessed by examining the Predicted Aligned Error (PAE) matrices and the per-residue predicted Local Distance Difference Test (pLDDT) scores.

The UniProtKB accession codes of the KCTD proteins used for AF3 modeling are as follows: KCTD1 (Q719H9); KCTD2 (Q14681); KCTD5 (Q9NXV2); KCTD6 (Q8NC69); KCTD8 (Q6ZWB6); KCTD10 (Q9H3F6); KCTD11 (Q693B1); KCTD12 (Q96CX2); KCTD13 (Q8WZ19); KCTD15 (Q96SI1); KCTD16 (Q68DU8); KCTD17 (Q8N5Z5); KCTD21 (Q4G0X4); TNFAIP1 (Q13829).

The residue ranges included in the predicted models were: KCTD8(205-322):KCTD12(206-325); KCTD8(205-322):KCTD16(162-280); KCTD12(206-325):KCTD16(162-280); KCTD11(14-271):KCTD21(4-260); KCTD6(12-234):KCTD11(14-271); KCTD2(72-239):KCTD5(42-211); KCTD2(72-239):KCTD17(30-196); KCTD5(42-211):KCTD17(30-196); KCTD10(33-260):KCTD13(41-271); KCTD10(33-260):TNFAIP1(28-257); KCTD13(41-271):TNFAIP1(28-257); KCTD5(154-211):KCTD8(205-322); KCTD5(154-211):KCTD16(162-280); KCTD1(30-239): KCTD11(14-271); KCTD1(30-239):KCTD21(4-260); KCTD15(56-265):KCTD21(4-260).

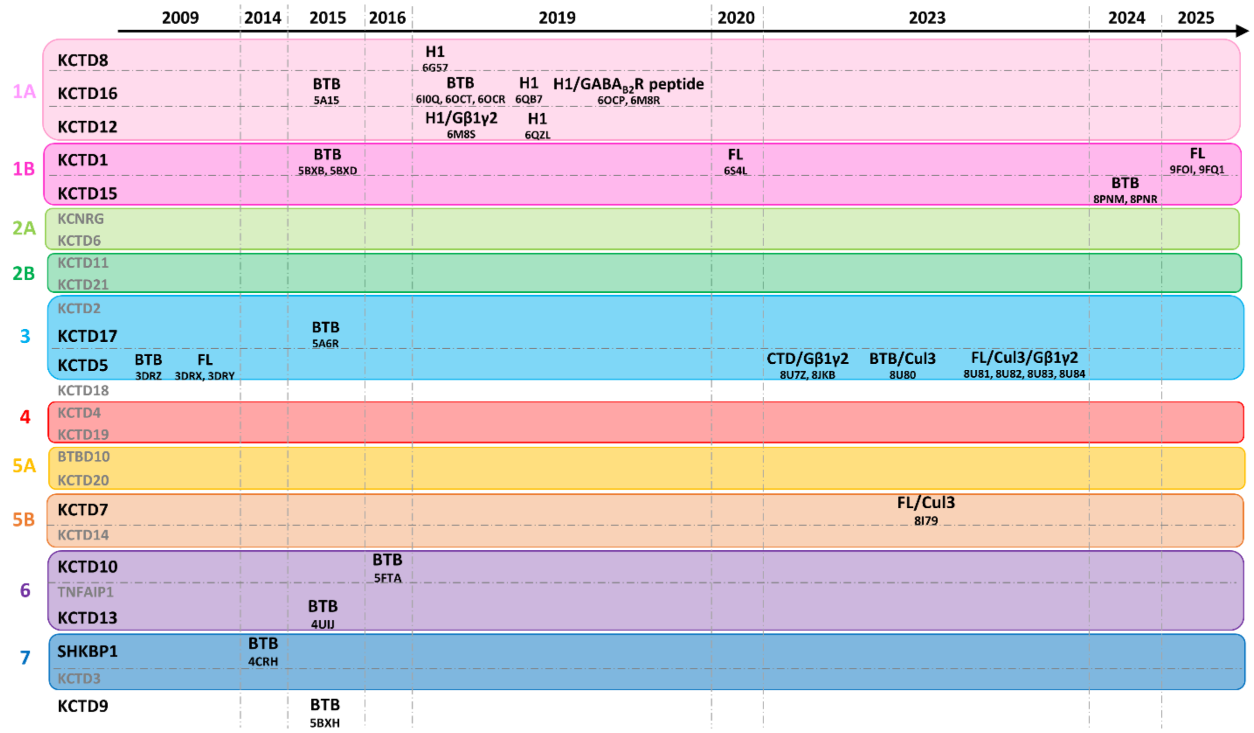

Figure S1. Chronological overview of the experimentally determined structures of KCTD protein family members, highlighting the temporal order in which structural data became available. For each structure, the protein domains, interacting partners, and corresponding PDB accession codes are indicated.

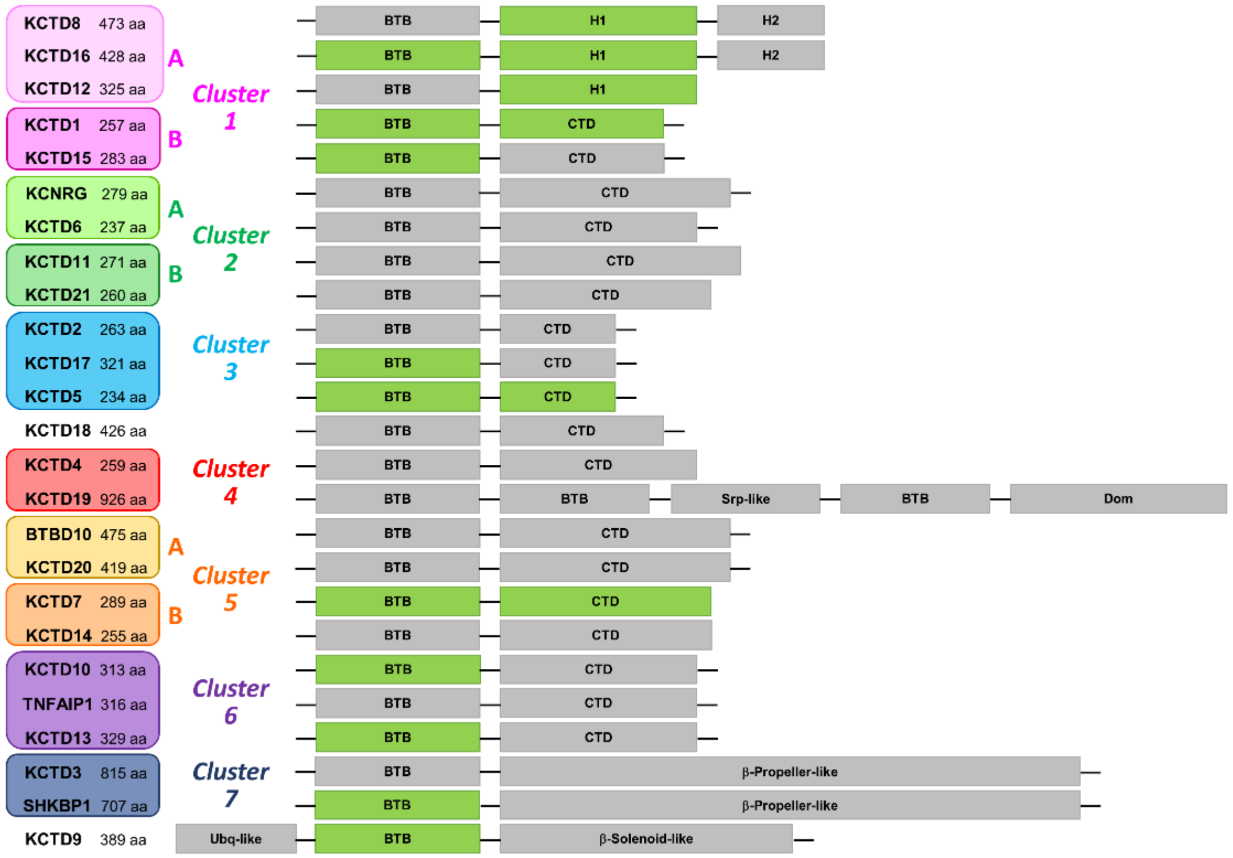

Figure S2. Overview of the experimental structural characterization of KCTD proteins. Domains with available experimentally determined structures (see Table 2) are in green, while domains for which no experimental structural information is currently available are shown in gray. For these latter proteins/domains, AF-predicted models have been reported (see main text – section 3).

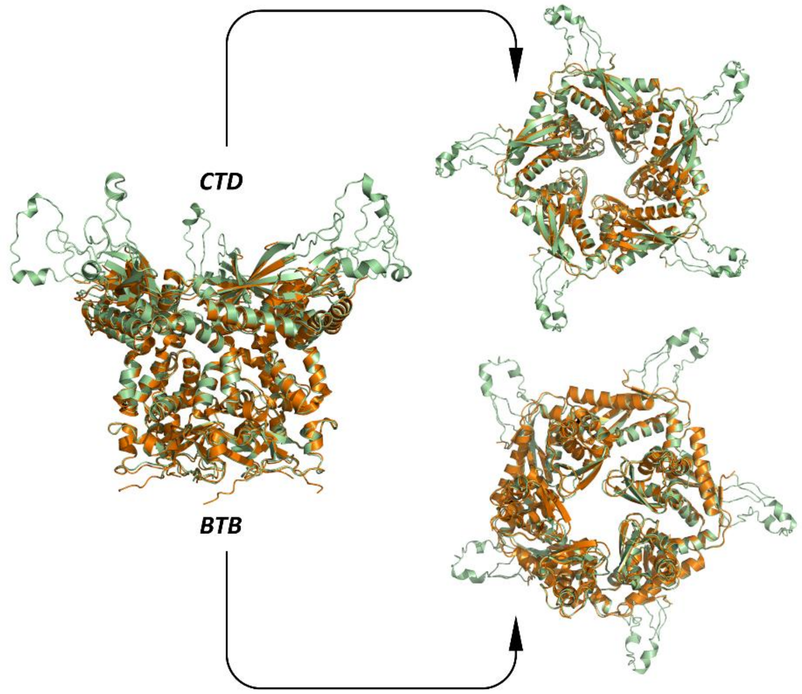

Figure S3. Structural superimposition of the experimental KCTD7 structure extracted from the KCTD7–Cul3 complex (PDB ID: 8I79; orange) and the AF-predicted KCTD7 structure (green). Different orientations of the superimposed structures are shown.

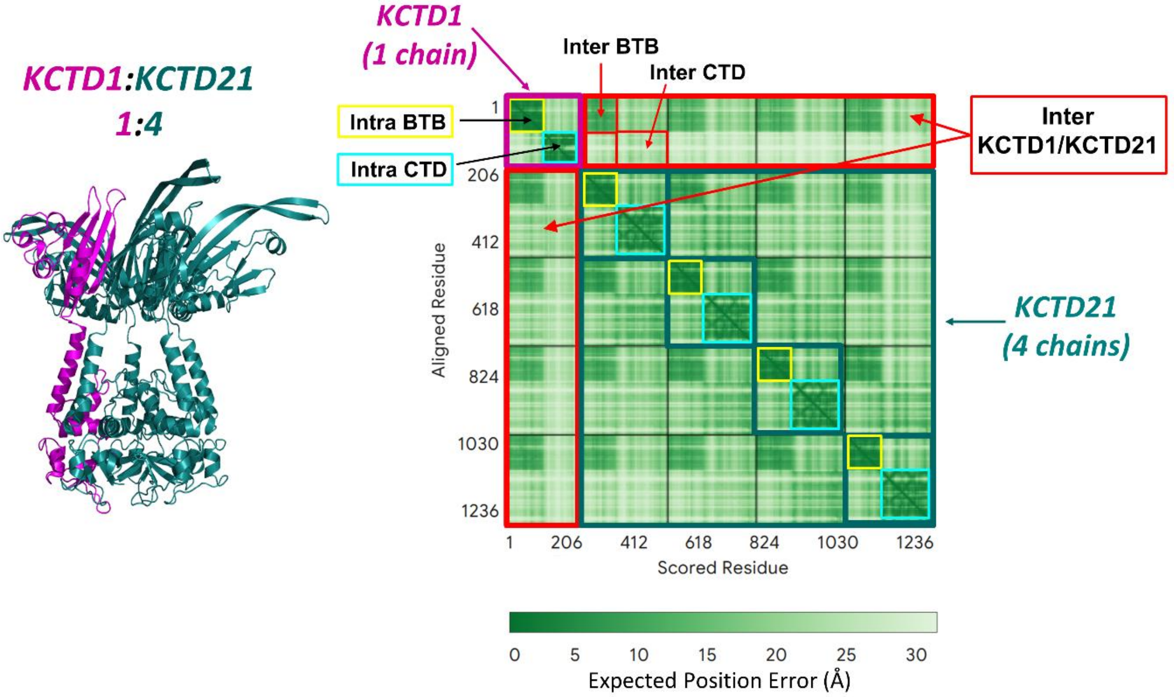

Figure S4. Example of a PAE matrix obtained for a KCTD hetero-complex (KCTD1:KCTD21 1:4). The regions corresponding to intra- and inter-molecular interactions are highlighted to illustrate the estimated prediction errors associated with each type of interaction.

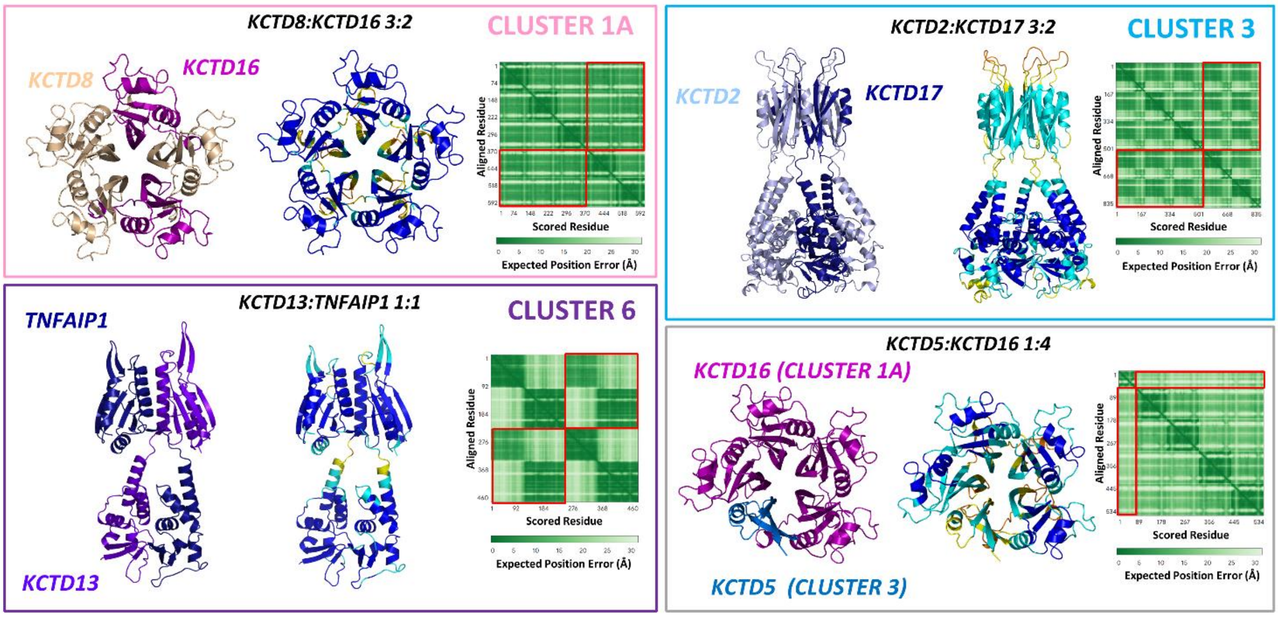

Figure S5. Cartoon representations of stable AF-predicted KCTD assemblies obtained by combining chains from two different KCTD family members. The models illustrate the structural arrangement of the resulting assemblies, with chains colored according to either their protein of origin or to the AF per-residue confidence metric (pLDDT) as follows: blue for pLDDT > 90, cyan for 70 < pLDDT ≤ 90, yellow for 50 < pLDDT ≤ 70, and orange for pLDDT < 50. Predicted Alignment Error (PAE) matrices are also shown; regions highlighted in red boxes indicate inter-chain interfaces between the different KCTD members.

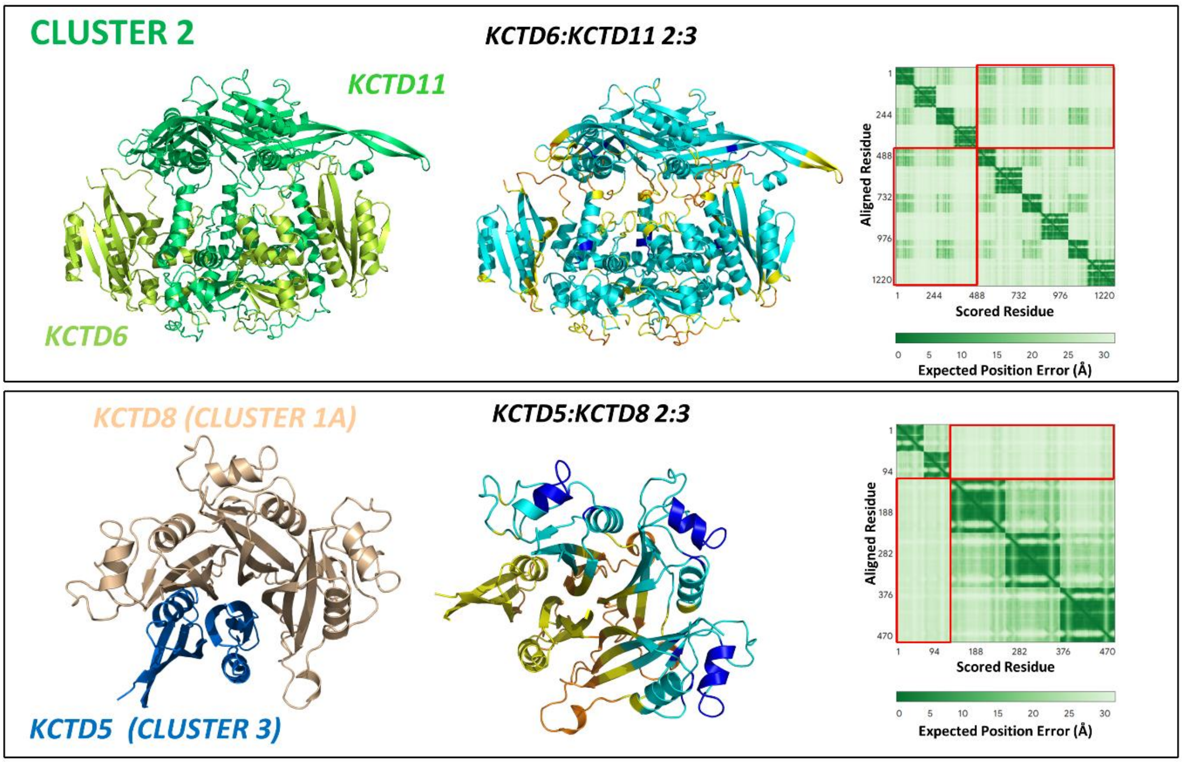

Figure S6. Cartoon representations of unstable AF-predicted KCTD assemblies obtained by combining chains from two different KCTD family members. The models illustrate the structural arrangement of the resulting assemblies, with chains colored according to either their protein of origin or to the AF per-residue confidence metric (pLDDT) as follows: blue for pLDDT > 90, cyan for 70 < pLDDT ≤ 90, yellow for 50 < pLDDT ≤ 70, and orange for pLDDT < 50. Predicted Alignment Error (PAE) matrices are also shown; regions highlighted in red boxes indicate inter-chain interfaces between the different KCTD members.
